# Electrophysiological Correlates of Familiarity and Recollection in Associative Recognition: Contributions of Perceptual and Conceptual Processing to Unitization

**DOI:** 10.3389/fnhum.2017.00125

**Published:** 2017-03-15

**Authors:** Bingcan Li, Xinrui Mao, Yujuan Wang, Chunyan Guo

**Affiliations:** ^1^Beijing Key Laboratory of Learning and Cognition, Department of Psychology, Capital Normal UniversityBeijing, China; ^2^Beijing Advanced Innovation Center for Imaging Technology, Capital Normal UniversityBeijing, China

**Keywords:** unitization, familiarity, recollection, perceptual and conceptual processing, associative recognition, ERPs (event-related potentials)

## Abstract

It is generally accepted that associative recognition memory is supported by recollection. In addition, recent research indicates that familiarity can support associative memory, especially when two items are unitized into a single item. Both perceptual and conceptual manipulations can be used to unitize items, but few studies have compared these two methods of unitization directly. In the present study, we investigated the effects of familiarity and recollection on successful retrieval of items that were unitized perceptually or conceptually. Participants were instructed to remember either a Chinese two-character compound or unrelated word-pairs, which were presented simultaneously or sequentially. Participants were then asked to recognize whether word-pairs were intact or rearranged. Event-related potential (ERP) recordings were performed during the recognition phase of the study. Two-character compounds were better discriminated than unrelated word-pairs and simultaneous presentation was found to elicit better discrimination than sequential presentation for unrelated word-pairs only. ERP recordings indicated that the early intact/rearranged effects (FN400), typically associated with familiarity, were elicited in compound word-pairs with both simultaneous and sequential presentation, and in simultaneously presented unrelated word-pairs, but not in sequentially presented unrelated word-pairs. In contrast, the late positive complex (LPC) effects associated with recollection were elicited in all four conditions. Together, these results indicate that while the engagement of familiarity in associative recognition is affected by both perceptual and conceptual unitization, conceptual unitization promotes a higher level of unitization (LOU). In addition, the engagement of recollection was not affected by unitized manipulations. It should be noted, however, that due to experimental design, the effects presented here may be due to semantic rather than episodic memory and future studies should take this into consideration when manipulating rearranged pairs.

## Introduction

Episodic memory supports the retrieval of information pertaining to previously experienced events. The processes engaged during episodic memory retrieval can be explained by dual-process models (Mandler, 1980), which propose that familiarity and recollection are the two main cognitive processes of recognition memory (Atkinson and Juola, 1974; Jacoby, 1991; Yonelinas, 2002; Yonelinas et al., 2010). Familiarity is a fast and automatic process that is defined by a general feeling of having encountered an event previously without recalling additional details. Recollection, on the other hand, is a slower process that refers to the retrieval of additional contextual details about the event, such as when and where an event took place.

Recent developments in event-related potential (ERP) studies provide evidence that familiarity and recollection processes are indexed by two qualitatively distinct components (Curran, 2000; Mecklinger, 2000, 2006; Rugg and Curran, 2007). An early bilateral frontal old/new effect (300–500 ms), often referred to as FN400, has been correlated with familiarity. By contrast, a late parietal old/new effect (500–800 ms), referred to as late positive component (LPC), has been correlated with recollection.

The ability to access episodic associations is important for the reconstruction of encountered environments. In the associative recognition paradigm, participants are presented with two or more items simultaneously. They are later asked to distinguish pairings of items previously presented together (e.g., intact pairs) from those not previously presented together (e.g., rearranged pairs). Conventionally, associative recognition is thought to be supported by recollection because judgments require the retrieval of an item and its context (Yonelinas, 1997; Donaldson and Rugg, 1998; Hockley and Consoli, 1999). However, recent studies have suggested that familiarity may also support associative memory, particularly if individuals treat the two items as a single unitized item (Yonelinas, 1999; Jäger et al., 2006; Quamme et al., 2007; Rhodes and Donaldson, 2007, 2008; Jäger and Mecklinger, 2009; Bader et al., 2010; Ahmad and Hockley, 2014; Zheng et al., 2015a). When unitized pairs are processed as a single item, such as the word pair “traffic-jam”, familiarity can support successful associative recognition for the combined item. The level of unitization (LOU) framework further proposes that there is a continuum of unitization such that any given pair of items may be processed more or less as two independent items or as a single item (Parks and Yonelinas, 2015). When individual elements cannot be unitized, recollection (instead of familiarity) must be used for retrieval. At a higher LOU, familiarity can support associative recognition. Therefore, a positive correlation exists between LOU and the degree to which familiarity can support associative memory (Parks and Yonelinas, 2015).

Various methods of item manipulation can affect LOU. Tibon et al. (2014b) identified two broad types of approaches to promote unitization: top-down and bottom-up. Top-down manipulations are based on a set of instructions to process pairs as a single unit, such as interactive imagery (i.e., the mental creation of an image of the two items interacting together) and definition encoding (Quamme et al., 2007; Rhodes and Donaldson, 2008; Shao et al., 2016). Bottom-up manipulations, on the other hand, are based on features of the presentation, or the inherent relationship between the paired items (Bastin et al., 2010; Tibon and Levy, 2014; Parks and Yonelinas, 2015). The bottom-up approach is a more natural method of unitization in that inherent or presentation-related features of items are perceptually or conceptually manipulated to increase LOU. Perceptual utilization includes changes in the spatiotemporal presentation of stimuli (simultaneous or sequential) or changes in stimulus modalities (Bastin et al., 2010; Tibon and Levy, 2014; Parks and Yonelinas, 2015). An example of temporal manipulations is as follows (Parks and Yonelinas, 2015): items are presented either sequentially, to ensure that the items are processed as two separate units, or simultaneously, to ensure that the two items are encoded as a single unit. During the retrieval process, sequentially presented pairs elicit less familiarity than simultaneously presented pairs. Modality relationships also affect unitization (Bastin et al., 2010). In a recent ERP study by Tibon et al. (2014a), participants were presented with either picture-picture pairs or picture-sound pairs. Associative recognition was accompanied by familiarity only for picture-picture pairs.

The LOU can also be manipulated by conceptual bottom-up approaches. The primary goal of these approaches is to manipulate the associative or semantic relationships between items, including compound words (Giovanello et al., 2006; Rhodes and Donaldson, 2007; Ahmad and Hockley, 2014), associative words (Opitz and Cornell, 2006), or semantic-related picture-pairs (Tibon et al., 2014b). For example, “traffic-jam” is an association word-pair in which the single words “traffic” and “jam” can be comprised into one unit. On the other hand, “piano-cereal” shares no semantic relationship and thus is not amenable to conceptual unitization. In an ERP study by Tibon et al. (2014b), participants were asked to remember related object picture-pairs (e.g., a lamp over a table) and unrelated object picture-pairs (e.g., a book over an apple). The results indicated that familiarity-associated early frontal old/new effects emerged only for related picture-pairs, providing evidence that semantic unitization can support associative familiarity (i.e., the familiarity elicited in associative recognition).

While prior studies have tested various means of enhancing unitization, few have compared two or more types of bottom-up approaches to unitization within the same experiment. While both conceptual and perceptual manipulations lead to associative familiarity, the two approaches may differ in their effectiveness. According to a levels of processing (LOP) framework, conceptual processing necessitates more semantic elaboration than perceptual processing and prior studies have shown that memory benefits more from semantic encoding than perceptual encoding (Craik and Lockhart, 1972; Craik and Tulving, 1975; Lockhart and Craik, 1990). Furthermore, compared to perceptual elaboration, semantic elaboration has a moderate effect on familiarity and a great effect on recollection (Yonelinas, 2001). Therefore, we hypothesized that a conceptually unitized task would lead to greater recollection-based associative recognition than a perceptually unitized task. In addition, we hypothesized that familiarity-based associative recognition would differ between conceptually unitized and perceptually unitized tasks.

The potential overlap between conceptual and perceptual manipulation makes a pure comparison between the two approaches difficult. Perceptual and conceptual representations are partially based on the same systems (Pecher et al., 2007; Van Dantzig et al., 2008). In the current study, perceiving a compound word-pair as a conceptual unit is influenced partly by its temporal presentation. However, as mentioned previously, perceptual and conceptual relationships are activated by different regions (Prince et al., 2005) and it may be argued that forming a conceptual unit is partly independent from perceptual unitization. The current study tested whether each manipulation approach (conceptual and perceptual) would contribute differently to the unitized familiarity effect.

In the present study, participants were asked to complete an associative recognition test after being presented with Chinese two-character compound and unrelated word-pairs, which were presented either simultaneously or sequentially. Previous studies have shown that compound word-pairs are conceptually unitized as a single item and elicit familiarity to support associative memory (Giovanello et al., 2006; Rhodes and Donaldson, 2007; Ford et al., 2010; Ahmad and Hockley, 2014). This phenomenon is reflected in ERP recordings by an early frontal intact/rearranged effect (i.e., a less negative deflection in response to previously encountered pairs relative to rearranged pairs). In the current study, participants were presented with either two-character compound words (high-conceptually unitized condition, HC) or unrelated word-pairs (low conceptually unitized condition, LC). Perceptual unitization was manipulated by altering the temporal presentation of stimuli without changing the modality of compound words. Thus stimuli were presented simultaneously in the high-perceptually unitized condition (HP), whereas stimuli were presented sequentially in the low-perceptually unitized condition (LP; Parks and Yonelinas, 2015).

An associative recognition task was used to test the study hypotheses. Participants were asked to learn word-pairs (e.g., A-B, C-D), and were later asked to discriminate between intact pairs (e.g., A-B) and rearranged pairs (e.g., A–C). Chinese two-character compound words were used for stimuli rather than traditional two-character word-pairs because the Chinese system is based on the association of morphemes with graphic units and one-character words can be perceptually unitized more easily with another word. During perceptual manipulations, the rearranged pairs were presented simultaneously during the testing phase of the study in accordance with the procedure described previously by Parks and Yonelinas (2015). The simultaneous presentation of word-pairs at retrieval permitted analysis of retrieval data and locked ERPs. For conceptual manipulation, all word-pairs were rearranged into unrelated word-pairs. ERP recordings were performed to investigate differences in response to intact, unitized word-pairs vs. in response to rearranged, unrelated word-pairs. However, if all of the rearranged pairs in the test phase were unrelated pairs, the compounds in the test phase would be easily recognized as intact. We therefore rearranged compounds and unrelated word-pairs into new compounds and added these new stimuli into the recognition test to minimize recognition judgments regarding whether the word-pairs were unitized or not.

Our first hypothesis was that the retrieval of simultaneously presented compound word-pairs (HP-HC) would be supported both by familiarity and recollection (Rhodes and Donaldson, 2007; Zheng et al., 2015b), indicated by intact/rearranged effects in both the FN400 and LPC time windows. The second hypothesis was that sequentially presented unrelated word-pairs (LP-LC) would lack an FN400, but maintain an LPC, because this condition should yield a low LOU. The third hypothesis was that conceptual manipulation of unitization would have a greater impact on the unitized familiarity and recollection effect than perceptual manipulation due to a higher LOP (Craik and Lockhart, 1972; Craik and Tulving, 1975). The sequential presentation of compound word-pairs (LP-HC) was expected to elicit a stronger difference in waveforms (intact minus rearranged) than simultaneous presentation of unrelated word-pairs (HP-LC) in both the FN400 and LPC time windows.

## Materials and Methods

### Participants

Twenty right-handed students from Capital Normal University participated in the experiment and were paid ¥30 per hour. Data from four participants were discarded due to insufficient artifact-free trials in the critical response categories. The mean age of the remaining 16 subjects was 22.31 years (range 19–25), with a male:female ratio of 6:10. All participants had normal or corrected to normal vision. The study was approved by The Human Research Ethics Committee of Capital Normal University. All subjects were informed about all aspects of the experiments and written informed consent was obtained from each participant on the day of the experimental session.

### Stimuli

The stimuli were comprised of 304 Chinese word-pairs (mean total number of strokes: 8.32 (range, 3–21), mean word frequency: 106.47 (range, 3.7–458.5) occurrences per million (Liu, 1990)). Stimuli included 120 compound word-pairs and 120 unrelated word-pairs without an associative or semantic relationship. Another 64 pairs composed of compound and unrelated word-pairs equally were added to the word-pairs as fillers. As mentioned above, the fillers were all rearranged into compound word-pairs during the recognition test. Word-pairs in each condition were matched for word frequency (mean: 106.47).

An independent sample of 20 native Chinese speakers (9 men and 11 women) participated in a pretest to determine the degree to which word-pairs in each condition could be treated as a single unit. Participants in the pretest were presented with word-pairs in a random order on a computer monitor. Participants were asked to answer the following: “determine how much you think that these word-pairs could be considered as a meaningful unit” using a scale ranging from 1 (hardly unitized) to 4 (completely unitized). Participants were informed that there was no correct answer and that making a subjective judgment was sufficient. The results confirmed that the set of compound word-pairs were rated as being more unitized (Mean = 3.89, SD = 0.06) than unrelated word-pairs (Mean = 1.27, SD = 0.100), *t*_(19)_ = 93.56, *p* < 0.001.

**Figure 1:**
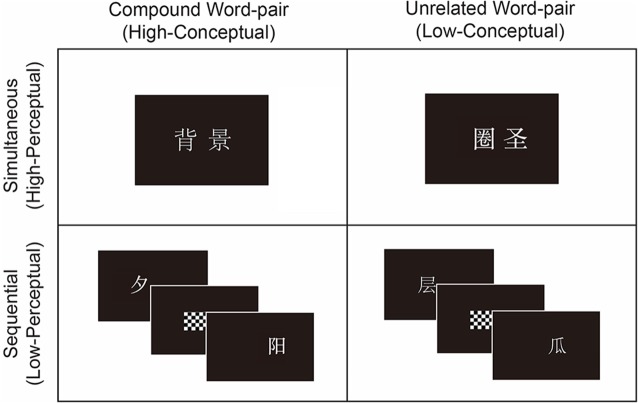
**Example stimuli.** In the high perceptual association condition, compound word-pairs (e.g., “
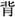

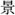
” meaning “back-ground”) and unrelated word-pairs (e.g., “
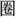
 
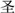
” meaning “circle-sage”) were presented simultaneously as high perceptual-high conceptual (HP-HC) and high perceptual-low conceptual (HP-LC) conditions respectively. In the low perceptual association condition, compound word-pairs (e.g., “
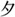
 
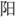
” meaning “sun-set”) and unrelated word-pairs (e.g., “
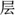
 
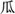
” meaning “layer-melon”) were presented sequentially as low perceptual-high conceptual (LP-HC) and low perceptual-low conceptual (LP-LC) conditions, respectively.

Word-pairs were presented in four study-test blocks, including two simultaneous blocks and two sequential blocks. All word-pairs were assigned with equivalent number of strokes and word frequency into the four blocks. The sequence of the four blocks was counterbalanced across participants. In each block, the study phase consisted of 30 compound word-pairs, 30 unrelated word-pairs and 16 filler word-pairs; the fillers included 8 compounds word-pairs and 8 unrelated word-pairs. In the simultaneous condition, the word-pairs were presented simultaneously, whereas in the sequential condition, another set of word-pairs were presented sequentially (see Figure 1). In the test phase, half of the studied word-pairs were recombined and presented as rearranged word-pairs with their original spatial locations unchanged. For example, “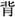-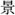
” and “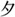-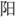
” are two Chinese compound word-pairs, meaning “back-ground” and “sun-set” respectively. After rearrangement, the presented word-pairs were “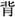-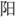
” and “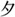-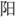
”. The test phase thus consisted of 30 intact word-pairs 30 rearranged word-pairs and 16 rearranged filler pairs in each block. Although we added fillers to minimize the possibility that participants would make recognition judgments based on whether word-pairs were unitized or not (see above), participants were also informed during the test phase that they should not make decisions based on whether the word-pairs were compounds, but rather by whether they thought the word-pairs were intact or rearranged.

### Procedure

Participants were seated in front of a 17-inch Dell computer monitor in an electrically shielded room and asked to remember word-pairs presented on the screen. Word-pairs in 18-point Simhei were displayed by Presentation software (Neurobehavioral Systems, San Francisco, CA, USA) on a computer monitor. Stimuli were displayed in white font against a black background. At a viewing distance of 70 cm, the stimuli subtended a maximum horizontal visual angle of 3.7° and a maximum vertical visual angle of 1.4°.

Prior to the study phase, subjects were instructed to remember word-pairs (compound word-pairs and unrelated word-pairs) for a later test. Instructions were presented on the computer screen and the experimenter explained and reiterated important points. Prior to commencing the task, each participant completed two practice blocks.

During the study phase, each trial began with the presentation of a fixation cross (+) in the center of the screen for 1000 ms after which the word-pairs were displayed on the screen. In the *simultaneous* condition, both words in the word-pair were presented on the screen for 1200 ms followed by a blank screen for 500 ms. In the *sequential* condition, the left-word was presented alone for 600 ms, followed by a checkerboard mask presented for 300 ms in the same location on the screen to prevent unitization of the two words (Parks and Yonelinas, 2015). After presentation of the mask, the right-word was presented alone for 600 ms, followed by a blank screen for 500 ms. Participants were told that the two words presented in the study phase would again be presented in the test phase and they would be either intact or rearranged. Thus, both compound and unrelated word-pairs should be remembered as a unit during the test. During the study phase, participants did not press any buttons. There was a 2-min gap between the study phase and the test phase.

During the test phase, each trial began with the presentation of a fixation cross for 1000 ms. All test word-pairs were presented simultaneously for 2000 ms followed by a blank screen for 1000 ms. During the test phase, participants were asked to make a judgment of whether the word-pair was the same as the test phase (“intact”) or whether the word was rearranged with another word (“rearranged”) using the F and J keys, respectively. Response hand assignment was counterbalanced across participants.

### Behavioral Data Analysis

To examine the influence of perceptual and conceptual manipulations on memory performance, accuracy results and response times (RTs) were each subjected to independent repeated measures analysis of variance (ANOVAs) with perceptual condition (simultaneous, sequential presentation), conceptual condition (compounds, unrelated word pairs) and response (intact, rearranged) as factors. Pr value (the proportion of hits in response to intact pairs minus the proportion of false alarms in response to rearranged pairs) were subjected to repeated-measures ANOVAs with perceptual condition (simultaneous, sequential presentation) and conceptual condition (compounds, unrelated word pairs) as factors. To analyze the discrimination ability of participants, associative Pr indices were submitted to a multivariate ANOVA (MANOVA).

### ERP Recording and Analyses

Scalp electroencephalogram (EEG) data were recorded from 62 Ag/AgCl electrodes embedded in an elastic cap equipped with a NeuroScan SynAmps system at a sampling rate of 500 Hz with a 0.05–100 Hz band pass filler. Eye movements were monitored by two electrodes placed at the outer canthi of each eye and one infra-orbitally placed electrode at the left eye. All voltages were referenced to the left mastoid online and re-referenced offline to the average of the left and right mastoid recordings. Impedance was less than 5 kΩ and EEG/electrooculogram (EOG) signals were digitally band pass filtered from 0.05 Hz to 40 Hz. Each averaging epoch lasted 1200 ms, beginning 200 ms prior to stimulus onset and corrected to a 200-ms pre-stimulus baseline. Trials with EEG voltages exceeding ±75 μV were discarded prior to averaging. EOG blink artifacts were corrected using a linear regression estimate (Semlitsch et al., 1986).

ERP data were collected and processed by Neuroscan software, and statistical analysis was performed using SPSS 20.0 statistical software. For each condition, a minimum of 18 analyzed trials was required. Repeated measures ANOVAs were conducted on mean amplitude relative to the 200-ms pre-stimulus baseline. Main effects and interactions are reported. To further investigate response, ANOVAs within each time window were conducted with perceptual condition, conceptual condition, response (intact, rearranged), location (anterior, central, posterior) and laterality (left, mid, right) as variables. Significant main or interaction effects were followed up with *post hoc* ANOVAs or paired *t*-tests. For each time window, the mean amplitudes of the intact/rearranged difference waveforms were then analyzed at each representative electrode to illustrate the magnitude of the intact/rearranged effects. To compare the magnitude of the old/new effects across conditions, an ANOVA on the difference in waveforms (intact minus rearranged) at Fz was conducted with perceptual and conceptual conditions as variables.

Finally, to examine whether qualitatively different configurations of neural generators were confounded by overall amplitude in different time windows, topographical analyses were performed on the intact and rearranged differences with vector-scaled data (McCarthy and Wood, 1985; Wilding, 2006). It is well accepted that differences in amplitude topography are mediated by distinct mechanisms (Allan et al., 2000).

## Results

### Behavioral Data

Behavioral task performance data are presented in Table 1.

**Table 1:** **The mean percentage (standard deviation) of correct responses and mean response time (RT) for the four conditions**.

Parameter	HP-HC		HP-LC		LP-HC		LP-LC	
	Intact	Rearranged	Intact	Rearranged	Intact	Rearranged	Intact	Rearranged
Pr	68.9 (18.1)		40.1 (17.9)		74.1 (17.8)		32.7 (16.1)	
Accuracy (%)	76.7 (12.4)	92.2 (6.9)	77.3 (10.0)	62.8 (14.5)	84.5 (10.3)	89.6 (9.7)	65.9 (11.1)	66.8 (12.0)
RT (s)	972 (147)	1056 (163)	1112 (187)	1237 (210)	954 (119)	1063 (145)	1173 (181)	1223 (186)

#### Accuracy

There was a main effect of conceptual condition (*F*_(1,15)_ = 136.06, *p* < 0.001, $\eta_{\text{p}}^{\text{2}}$ = 0.90), an interaction between conceptual condition and response (*F*_(1,15)_ = 12.35, *p* < 0.005, $\eta_{\text{p}}^{\text{2}}$ = 0.45), and a three-way interaction between perceptual condition (simultaneous, sequential presentation), conceptual condition (compounds, unrelated word pairs), and response (intact, rearranged; *F*_(1,15)_ = 36.76, *p* < 0.001, $\eta_{\text{p}}^{\text{2}}$ =0.71) on accuracy. Compounds were recognized more accurately than unrelated word-pairs (*p* < 0.001).

For intact responses, *post hoc* analysis revealed a main effect of conceptual condition (*F*_(1,15)_ = 6.93, *p* < 0.05, $\eta_{\text{p}}^{\text{2}}$ = 0.32) and a significant interaction between perceptual condition and conceptual condition (*F*_(1,15)_ = 57.92, *p* < 0.001, $\eta_{\text{p}}^{\text{2}}$ = 0.79) on accuracy. Similar to the above result, compounds were better recognized than unrelated word-pairs (*p* < 0.05). Pair-wise comparisons revealed a significant increase in the hit rate for compound word-pairs compared to unrelated word-pairs in the sequential presentation (*t*_(15)_ = 4.63, *p* < 0.05), but not in the simultaneous presentation condition (*t*_(15)_ = 0.195, *p* > 0.05).

For rearranged responses, *post hoc* analysis revealed a main effect of conceptual condition (*F*_(1,15)_ = 141.12, *p* < 0.001, $\eta_{\text{p}}^{\text{2}}$ = 0.90) and a significant interaction between perceptual condition and conceptual condition (*F*_(1,15)_ = 5.26, *p* < 0.05, $\eta_{\text{p}}^{\text{2}}$ = 0.26). Compounds were again rejected with greater accuracy than unrelated word pairs (*p* < 0.001). Pair-wise comparisons revealed a significant higher correct rejection rate for compound word-pairs than for unrelated word-pairs in both simultaneous and sequential presentation conditions (*t*_(15)_ = 11.89, *p* < 0.001; *t*_(15)_ = 8.26, *p* < 0.001).

#### Associative Pr

A MANOVA revealed a main effect of conceptual condition (*F*_(1,15)_ = 136.06, *p* < 0.001, $\eta_{\text{p}}^{\text{2}}$ = 0.90), and a significant interaction between perceptual condition and conceptual condition (*F*_(1,15)_ = 14.39, *p* < 0.005, $\eta_{\text{p}}^{\text{2}}$ = 0.49), on Pr value. Pair-wise comparisons revealed that discrimination was significantly better for compounds than for unrelated word-pairs (*t*_(15)_ = 11.66, *p* < 0.001). The Pr value for unrelated word-pairs was higher for simultaneous presentation than for sequential presentation (*t*_(15)_ = 1.99, *p* = 0.065). However, for compounds, there no significant differences in Pr value (*t*_(15)_ = 1.68, *p* = 0.113).

#### RT

Compound word-pairs elicited faster RTs than unrelated word-pairs across response types (see Table 1). A repeated measures ANOVA on RTs revealed a main effect of conceptual condition (*F*_(1,15)_ = 69.81, *p* < 0.01, $\eta_{\text{p}}^{\text{2}}$ = 0.84), a main effect of response (*F*_(1,15)_ = 31.75, *p* < 0.001, $\eta_{\text{p}}^{\text{2}}$ = 0.68), and a three-way interaction of perceptual condition, conceptual condition, and response (*F*_(1,15)_ = 12.00, *p* < 0.005, $\eta_{\text{p}}^{\text{2}}$ = 0.44). Compounds were reacted to faster than unrelated word-pairs (*p* < 0.001). For intact responses, further analyses revealed a main effect of conceptual condition (*F*_(1,15)_ = 46.89, *p* < 0.001, $\eta_{\text{p}}^{\text{2}}$ = 0.76) and a significant interaction between perceptual condition and conceptual condition (*F*_(1,15)_ = 12.59, *p* < 0.005, $\eta_{\text{p}}^{\text{2}}$ = 0.46). Compounds were recognized faster than unrelated word-pairs (*p* < 0.001). Pair-wise comparisons showed that RTs to unrelated word-pairs were faster for simultaneous presentation than for sequential presentation (*t*_(15)_ = 3.14, *p* < 0.01). There were no significant differences in RT to compound word-pairs between simultaneous and sequential presentation (*t*_(15)_ = 1.21, *p* > 0.05). For rearranged responses, further analysis revealed a significant main effect of conceptual condition only (*F*_(1,15)_ = 76.31, *p* < 0.001, $\eta_{\text{p}}^{\text{2}}$ = 0.84), with compounds being correctly rejected faster than unrelated word-pairs (*p* < 0.001).

### ERP Data

#### ERP Analyses

**Figure 2:**
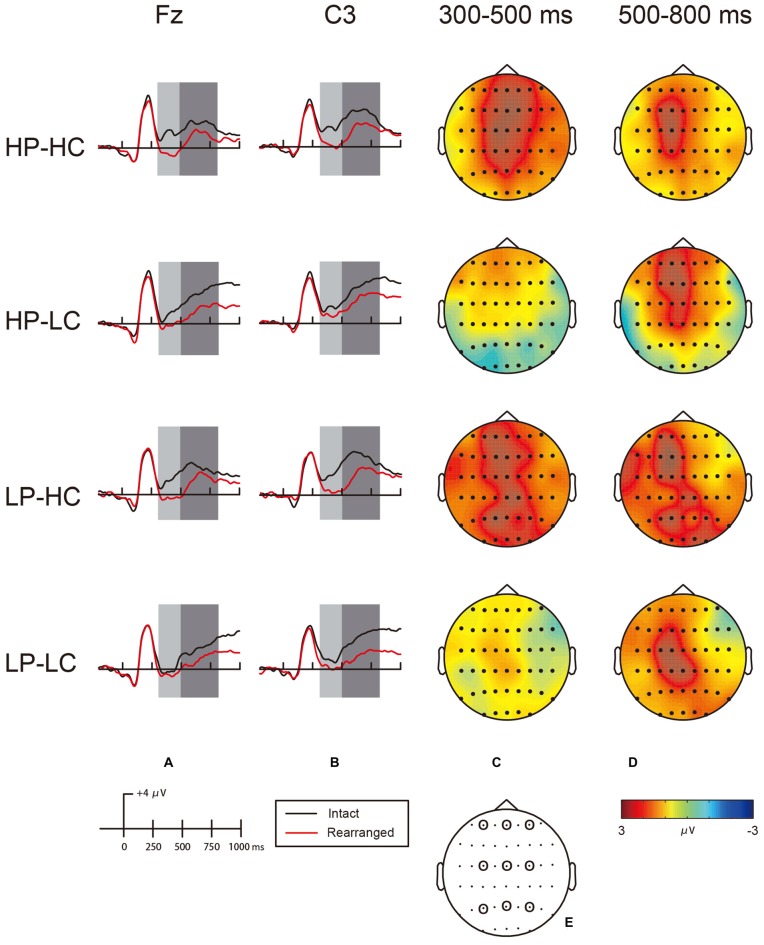
**(A)** Event-related potential (ERP) waveform for HP-HC, HP-LC, LP-HC and LP-LC at electrodes Fz in the 300–500-ms early time window (light bars) and the 500–800-ms time window (dark bars). **(B)** ERP waveform for HP-HC, HP-LC, LP-HC and LP-LC at electrodes C3 in the 300–500-ms early time window (light bars) and the 500–800-ms time window (dark bars). **(C)** Topographic maps illustrating the distribution of associative recognition differences (intact minus rearranged trials mean amplitudes) for HP-HC, HP-LC, LP-HC and LP-LC conditions during the 300–500-ms early time window. **(D)** Topographic maps illustrating the distribution of associative recognition differences (intact minus rearranged trials mean amplitudes) for HP-HC, HP-LC, LP-HC and LP-LC conditions during the 500–800-ms late time window. The scale bar in the bottom of the map indicates the maximum and minimum of the voltage range. **(E)** Schematic maps with the highlighted sites employed to analyze the intact/rearranged effects of 300–500-ms and 500–800-ms time windows. LF, left frontal (F3); MF, middle frontal (Fz); RF, right frontal (F4); LC, left central (C3); MC, middle central (Cz); RC, right central (C4); LP, left parietal (P3); MP, middle parietal (Pz); RP, right parietal (P4).

The grand average ERPs of correct responses to intact and rearranged word-pairs at frontal and central sites are depicted in Figure 2. As shown, the ERPs in response to intact word-pairs become more positive than ERPs in response to rearranged word-pairs approximately 300 ms after stimulus onset. Based on visual inspection and previous research (Tsivilis et al., 2001; Wolk et al., 2006; Rugg and Curran, 2007; Speer and Curran, 2007), the data were divided into two consecutive time windows, 300–500 ms (early) and 500–800 ms (late), to characterize the early bilateral frontal and late parietal effects, respectively. In each time window, the mean amplitudes were obtained from three frontal (F3, Fz, F4), three central (C3, Cz, C4) and three parietal (P3, Pz, P4) electrodes (Bader et al., 2010; Zheng et al., 2016).

Based on visual inspection of topographic maps, early intact/rearranged effects (typically associated with familiarity) were greatest during the early time window at frontal electrodes for both the HP and HC conditions. However, the ERPs were more broadly distributed for the HC condition. Intact/rearranged differences were more widely distributed in all conditions, and greatest at left central sites.

#### Early Time Window (300–500 ms)

There was a main effect of response (*F*_(1,15)_ = 82.87, *p* < 0.001, $\eta_{\text{p}}^{\text{2}}$ = 0.85), a conceptual condition × response interaction (*F*_(1,15)_ = 10.15, *p* < 0.01, $\eta_{\text{p}}^{\text{2}}$ = 0.40) and a perceptual condition × response × location interaction (*F*_(2,30)_ = 5.94, *p* < 0.01, $\eta_{\text{p}}^{\text{2}}$ = 0.28) during the 300–500 ms early time window. The amplitudes of intact response were significantly higher than those in rearranged response (*p* < 0.001). To further explore the three-way interaction, separate analyses were conducted for the HP condition and the LP condition collapsed across location and conceptual conditions. In the HP analysis, a significant interaction of response × location was found (*F*_(2,30)_ = 9.86, *p* = 0.01). The interaction showed a significantly larger difference between intact and rearranged pairs at frontal (*F*_(1,15)_ = 77.25, *p* < 0.001) compared to central (*F*_(1,15)_ = 35.93, *p* < 0.001) and parietal (*F*_(1,15)_ = 13.26, *p* < 0.005) sites. In the LP analysis, there was a main effect of response (*F*_(1,15)_ = 24.4, *p* < 0.001), with intact word-pairs eliciting higher amplitudes than rearranged word-pairs (*p* < 0.001).

The Fz electrode was chosen to represent bilateral frontal effects in each condition based on visual inspection and previous research (Bader et al., 2010; Zheng et al., 2016). An ANOVA conducted with perceptual condition, conceptual condition and response as variables revealed a main effect of response (*F*_(1,15)_ = 120.68, *p* < 0.001, $\eta_{\text{p}}^{\text{2}}$ = 0.89) and an interaction of conceptual condition × response (*F*_(1,15)_ = 17.73, *p* = 0.05, $\eta_{\text{p}}^{\text{2}}$ = 0.23). Although the perceptual condition × conceptual condition × response interaction was not significant (*F*_(1,15)_ = 0.3, *p* = 0.59, $\eta_{\text{p}}^{\text{2}}$ = 0.02), planned comparisons were carried out to directly analyze the intact/rearranged effects in each condition. The results confirmed our hypothesis that the ERP amplitudes in response to intact word-pairs were significantly higher than in response to rearranged word-pairs in the HP-HC, the LP-HC and the HP-LC conditions (*t*_(15)_ = 5.1, *p* < 0.001; *t*_(15)_ = 4.67, *p* < 0.001; *t*_(15)_ = 2.78, *p* < 0.05, respectively) but not in the LP-LC condition (*t*_(15)_ = 1.51, *p* > 0.05).

An ANOVA of the difference in waveforms (intact minus rearranged) at Fz conducted with perceptual and conceptual conditions as variables revealed a significant main effect of conceptual condition (*F*_(1,15)_ = 4.45, *p* = 0.05, $\eta_{\text{p}}^{\text{2}}$ = 0.23), indicating that word-pairs in the HC condition elicited greater differences in waveforms than word-pairs in the LC condition. Although the interaction between perceptual and conceptual conditions was not significant, planned *t*-test comparisons were conducted. Results showed that the amplitude differences in the LP-HC condition were significantly higher than those in LP-LC condition (*t*_(15)_ = 2.47, *p* < 0.05), but amplitude differences in the HP-LC condition were not significantly different than those in LP-LC condition (*t*_(15)_ = 1.28, *p* > 0.05). In sum, the analyses for the early time window revealed a reliable frontal intact/rearranged effect in the HP-HC, the LP-HC and the HP-LC conditions, but not in the LP-LC condition.

#### Late Time Window (500–800 ms)

The initial ANOVA for the late time window revealed a main effect of response (*F*_(1,15)_ = 64.52, *p* < 0.001, $\eta_{\text{p}}^{\text{2}}$ = 0.81), and interactions for response × laterality (*F*_(2,30)_ = 8.2, *p* < 0.005, $\eta_{\text{p}}^{\text{2}}$ = 0.81), perceptual condition × response × location (*F*_(2,30)_ = 4.77, *p* < 0.05, $\eta_{\text{p}}^{\text{2}}$ = 0.24) and response × location × laterality (*F*_(2,30)_ = 3.77, *p* < 0.01, $\eta_{\text{p}}^{\text{2}}$ = 0.20). The amplitudes of intact response were significantly higher than those in rearranged response (*p* < 0.001). To further investigate the three-way interaction of response × location × laterality, separate analyses at nine electrodes collapsed across perceptual and conceptual conditions were run. There was a significant difference between intact and rearranged word-pairs at all electrodes (*ps* < 0.005) with the greatest difference found at C3 (*F*_(1,15)_ = 66.26, *p* < 0.001), showing the maximal location was at left-central sites.

Due to the above results and previous researches (Bader et al., 2010; Zheng et al., 2015b), the electrode at C3 was chosen as the representative electrode for the late time window analysis. An ANOVA conducted with perceptual condition, conceptual condition and response (intact, rearranged) as variables revealed a significant main effect of response (*F*_(1,15)_ = 66.26, *p* < 0.001, $\eta_{\text{p}}^{\text{2}}$ = 0.82), with responses to intact word-pairs eliciting a higher amplitude than rearranged word-pairs. There was no significant interaction related to the conceptual or perceptual conditions indicating that the distribution of late intact/rearranged effects was not affected by perceptual or conceptual manipulations. An ANOVA conducted on difference waveforms at C3 in the late time window with perceptual and conceptual conditions as variables showed no significant main effects or interactions of perceptual and conceptual conditions on the difference waveforms at C3 (*F*_(1,15)_ = 1.25, *p* = 0.28, $\eta_{\text{p}}^{\text{2}}$ = 0.08), indicating that the late intact/rearranged effect sizes in the four conditions did not significantly differ. In sum, analyses on the late time window showed broadly distributed late intact/rearranged effects in the four conditions, which were greatest at C3. Manipulation of perceptual and conceptual conditions did not affect the distribution.

#### Topographic Analysis

Topographical analyses of the intact and rearranged differences using vector-scaled data for the 300–500-ms vs. the 500–800-ms time window revealed a significant interaction between time window and location (*F*_(61,915)_ = 2.71, *p* < 0.05, $\eta_{\text{p}}^{\text{2}}$ = 0.08), indicating different topographic distributions between early and late old/new effects. The early old/new effects were distributed more anteriorly while the late old/new effects were distributed in the left central site.

## Discussion

The current study investigated the unitization familiarity and recollection effect by manipulating perceptual and conceptual approaches. The behavioral results indicated that recognition performance was affected by both perceptual and conceptual approaches to unitization. Whether presented simultaneously or sequentially, compound word-pairs were better recognized than unrelated word-pairs according to Pr value. The RT to compound word-pairs was faster than the RT to unrelated word-pairs. In addition, simultaneous presentation elicited better associative discrimination and faster RT than sequential presentation in unrelated word-pairs but not in compound word-pairs. ERP results showed a significant intact/rearranged effect in the early time window in the HP-HC, HP-LC and LP-HC conditions, but not in the LP-LC condition. In the early time window, associative recognition differences in the LP-HC condition were significantly greater than those in the LP-LC condition. However, the differences in the HP-LC condition did not differ significantly from those in the LP-LC condition. The distributions were more posterior and widely distributed in the HP-HC and LP-HC conditions compared to the HP-LC condition. In the late time window, left central intact/rearranged effects, interpreted as recollection, were observed in all conditions. Moreover, the same pattern of difference waveforms of associative recognition was observed in all four conditions.

### The Early Frontal Intact/Rearranged Effect

In the early time window (300–500 ms), there was a significant intact/rearranged effect in the HP-HC, HP-LC and LP-HC conditions but not in the LP-LC condition, indicating that word-pairs without any unitized manipulation did not elicit associative familiarity. However, perceptual or conceptual approaches alone did elicit associative familiarity. Many others have described an increase in associative familiarity with the use of either perceptually or conceptually enhanced relationships (Ahmad and Hockley, 2014; Zheng et al., 2015a). In a behavioral study using image-sound pairs sound, Parks and Yonelinas (2015) found that simultaneous presentation increased familiarity compared to sequential presentation.

Interestingly, there was no difference in FN400-elicited strength between simultaneous and sequential presentation of compound word-pairs. Simultaneous presentation of unrelated word-pairs (HP-LC) however, did enhance the contribution of familiarity to associative retrieval. It may be that the strength of a compound word-pair for unitization can overcome the deficit produced by sequential presentation. On the other hand, the unitization of unrelated word-pairs was improved by perceptual association. The LOU framework states that there is a continuum of unitization such that any given pair of items may be processed more or less as independently or together (Parks and Yonelinas, 2015). Compared with low-level unitization, high-level unitization elicits greater familiarity with a more significant old/new effect. In the current study, the perceptual or conceptual approach alone was sufficient to elicit associative familiarity. However, conceptual unitization had a stronger effect on associative familiarity than perceptual unitization, reflected by a significant intact/rearranged difference between LP-HC and LP-LC, but no significant intact/rearranged difference between HP-LC and LP-LC. It appears that, at least in the current study, the conceptual approach elicits a higher LOU than the perceptual approach.

Both compound and unrelated word-pairs elicited an early intact/rearranged effect when presented simultaneously. However, Zheng et al. (2015b) found that the familiarity-related ERP effect was abolished if an unrelated word-pair was presented simultaneously. The difference between the current results and those presented by Zheng et al. (2015b) may be due to the stimuli used. Zheng et al. (2015b) used word-pairs composed of two-character Chinese words (e.g., 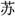

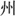
-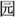

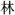
), whereas one-character Chinese word pairs (e.g., 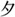-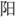) were used as units in the current study. Obviously, the one-character word pairs (two characters total) demand less attention, and are more easily unitized conceptually and perceptually during encoding relative to the two-character word-pairs (four characters total). Therefore, the properties of the stimuli to be unitized are a factor affecting LOU.

According to our topographic maps, word-pairs in the HP-LC condition elicited a standard early bilateral frontal intact/rearranged effect, whereas the LP-HC and HP-HC conditions elicited a more posteriorly distributed effect, similar to that reported by Bader et al. (2010), who suggested that the parietal old/new effect, not the earlier onset of late parietal old/new effect, reflects familiarity. Topographical analyses in the current study revealed that the early and late effects differ with respect to the configuration of underlying neural generators. Thus, our results are consistent with the assumption of Bader et al. (2010) and provide evidence supporting the suggestion that the parietal old/new effect indeed reflects familiarity.

The posterior and wide distribution elicited by compound word-pairs in the current study closely resembles the N400 component, which has been associated previously with ease of semantic integration (Olichney et al., 2000; Wolk et al., 2004). Compared to unrelated word-pairs, compound word-pairs carry greater conceptual fluency and are more likely to be judged as familiar (Whittlesea and Williams, 2001). Even when presented sequentially, compound word-pairs can be retrieved by an associative relationship based on greater conceptual fluency eliciting an N400 component relative to conceptual processing.

Voss and Paller (2007) argued that the old/new frontal effect may be related to priming rather than familiarity (Voss and Paller, 2006, 2007; Paller et al., 2007; Voss et al., 2010). As mentioned above, the intact/rearranged effect for compound word-pairs resembled the N400 component, presumably because compound word-pairs were based on greater conceptual fluency. However, Paller et al. (2007) proposed that in tests of recognition, previously presented stimuli were more conceptually fluent than newly presented pairs. According to this conceptual priming account, the presentation of word-pairs during the study phase in the current study may have facilitated conceptual processing of the word-pairs in the test phase. The conceptual priming account should therefore predict that the old/new effect was present for all conditions, or only in the high conceptual conditions. In the current study, the intact/rearranged effect was present in the HP-HC, HP-LC and LP-HC conditions but not the LP-LC condition, which is inconsistent with this theory. Therefore, whether the effects in the early time window found in the current study can be interpreted as conceptual priming warrants further investigation.

### The Late Left-Central Intact/Rearranged Effect

In the late ERP time window, an LPC appeared in all four conditions with no significant differences in amplitude. These results indicate that the contribution of familiarity to recognition of associated word-pairs was not accompanied by a corresponding reduction in recollection. These results are consistent with those of Zheng et al. (2015b) who reported that compared to unrelated word-pairs, recognition for compounds is reflected in both FN400 and an LPC, providing evidence that both familiarity and recollection processes enhance associative recognition when items are unitized. However, this observation is inconsistent with results presented by Yonelinas (2001) indicating conceptual but not perceptual effects on recollection.

In addition, the late intact/rearranged effect in the present study was greatest in the left-central electrodes, which is more widely distributed than the commonly observed left parietal old/new effect. However, ERP studies on associative memory have shown that the left-central location reflects recollection (Bader et al., 2010; Pilgrim et al., 2012; Zheng et al., 2015b).

### Familiarity and Recollection as Two Independent Processes

In the current study, perceptual and conceptual unitization was found to affect the contribution of familiarity processes to associative recognition, but not the contribution of recollection processes. Using a compound task and an imagery task to unitize word-pairs, Shao et al. (2016) found increased recollection and decreased familiarity for unitized word-pairs. They further proposed that, in an associative recognition task, increased recollection reduces the contribution of familiarity. The current results, however, do not support this idea because we found that unitization affected familiarity but not recollection. On the contrary, Tibon and Henson (2015) claimed that increased familiarity reduced the contribution of recollection in an associative task. In agreement, using a compound task to promote unitization, Bader et al. (2010) found that unitized word-pairs elicited an FN400 component and that only non-unitized word-pairs elicited an LPC. Bader et al. (2010) further proposed that familiarity was sufficient to support associative recognition and that recollection should not be required for remembering new concepts. However, in the current study, our findings suggested that recollection was not affected by a contribution of familiarity. Therefore, the current study supports the traditional dual-process theory that familiarity is independent from recollection.

However, the appearance of an LPC in high unitization conditions contrasts with the results of a study by Jäger et al. (2006). In the Jäger et al. (2006) study, participants were asked to remember pairs of faces representing two different people (inter-item condition) or pairs of two physically different but still very similar faces (intra-item condition). ERP results indicated that in the intra-item condition, familiarity alone was sufficient to support recognition and recollection was not necessary. It may be that the unitized item-pairs were very similar and as such retrieval demanded little information. Vilberg et al. (2006) investigated the electrophysiological correlates of recollection and found that the magnitude of the left parietal old/new effect was proportional to the amount of information recollected. In the current study, the compound word-pairs were constructed from two different concepts so that the retrieval necessitated a larger amount of information. Therefore, sufficient recollection was also essential for retrieval of perceptually and conceptually unitized information. In addition, the current results provide evidence that the amplitude of the LPC does not differ between the two conditions, which is inconsistent with our hypothesis that there would be a greater LPC for pairs in the LP-HC condition than in the HP-LC condition. It may be that the large amount of information necessary for retrieval did not differ between conditions and, as such, that a strong LPC was revealed in both conditions.

### Limitations

The current study poses some notable limitations. First, in the typical associative recognition task using compounds to manipulate unitization, the rearranged pair is also a compound word (Giovanello et al., 2006; Ahmad and Hockley, 2014). In the current study, however, the compounds were rearranged into unrelated word-pairs. This procedure may have facilitated the recognition of rearranged word-pairs by enabling recall that part of the pair was from a previously encountered compound word-pair. The alteration of the semantic link at retrieval may indicate that the intact/rearranged effects in ERP results can be attributed to semantic, rather than episodic, memory. As we observed in the early time window, the more posterior topographic distribution of the early old/new effect may not be the FN400 component, but rather the N400 component. Therefore, episodic and semantic effects cannot be easily disentangled. To avoid this confusion in future studies, words composing a compound word-pair during the study phase should be rearranged into compound word-pair during the test phase.

Second, at retrieval, all word-pairs were presented simultaneously. Therefore, stimuli were presented in the same manner at encoding and retrieval in the HP condition (all simultaneous), but not in the LP condition (encoding was sequential but retrieval was simultaneous). Therefore, the intact/rearranged effect in the LP condition may be due to a presentation mismatch at retrieval rather than perceptual unitization at encoding. The presentation of perceptual context at encoding and retrieval should remain consistent in future studies.

We rearranged compounds and unrelated word-pairs into new compounds and added these new stimuli as fillers into the recognition test to minimize the possibility that participants would make recognition judgments based on whether word-pairs were unitized or not. However, this manipulation made the number of intact and rearranged word-pairs unequal. In the current behavioral results, the average accuracy for a hit in all conditions was 76.1% and the accuracy for a correct rejection was 77.9%. These results indicate that the difference in proportion of intact and rearranged word-pairs did not affect participant bias. However, because the fillers were all rearranged into unrelated word-pairs and the proportions of compound and unrelated word-pairs were not equal at testing, we cannot rule out the possibility that the unequal proportions of compound and unrelated word-pairs could have affected participant bias. Future studies should be more cautious with filler manipulation.

In the early time window, the interaction between the perceptual and conceptual conditions on the difference in waveforms was not significant. Planned *t*-tests, however, revealed significant differences. It is therefore surprising that there was no significant two-way interaction. Given the mean amplitude differences (± standard deviations) observed in each condition (HP-HC, 2.74 ± 2.15; HP-LC, 1.51 ± 2.17; HP-HC, 2.45 ± 2.10; HP-HC, 0.71 ± 1.88), it appears that the non-significance of the interaction may be due to the relatively large standard deviations.

Finally, the ERP late intact/rearranged effect in the current study was greatest at the left-central location. In previous studies (Jäger et al., 2006; Rhodes and Donaldson, 2008), however, the effect was greatest at posterior locations. The reason for the difference in findings between the present and former experiments is unclear and warrants further investigation.

In summary, the current study provides evidence that a perceptual or conceptual approach to unitization alone is sufficient to evoke familiarity in associative recognition based on materials used in the present study. Conceptual unitization, however, elicits a higher LOU than perceptual unitization. Additionally, the contribution of recollection processes to associative recognition is not affected by unitized manipulations.

## Author Contributions

BL co-designed the experiment, collected and analyzed the behavioral and ERP data and co-wrote the text. XM co-designed the experiment, gave advise on the analyses of the data and co-wrote the text. YW gave advice on the analyses of the results and co-wrote the text. CG co-designed the experiment, advised on many aspects of the research and co-wrote the text.

## Conflict of Interest Statement

The authors declare that the research was conducted in the absence of any commercial or financial relationships that could be construed as a potential conflict of interest.
